# Formen der Evidenzsynthese

**DOI:** 10.1007/s00120-021-01476-x

**Published:** 2021-03-03

**Authors:** S. Graf, J. Kranz, S. Schmidt, L. Bellut, A. Uhlig

**Affiliations:** 1 Klinik für Urologie und Andrologie, Kepler Universitätsklinikum Linz, Krankenhausstraße 9, 4020 Linz, Österreich; 2 UroEvidence, Deutsche Gesellschaft für Urologie, Berlin, Deutschland; 3 Klinik für Urologie und Kinderurologie, St.-Antonius Hospital GmbH, Eschweiler, Deutschland; 4 Universitätsklinik und Poliklinik für Urologie, Universitätsklinikum Halle (Saale), Halle (Saale), Deutschland; 5 Klinik für Urologie und Kinderurologie, Uniklinik Erlangen, Erlangen, Deutschland; 6 Klinik für Urologie, Universitätsmedizin Göttingen, Göttingen, Deutschland

**Keywords:** Evidenzbasierte Medizin, Reviews, Bias, Confounder, Metaanalysen, Evidence-based medicine, Systematic reviews as topic, Bias, Confounding variables, Meta-analyses

## Abstract

Der vorliegende Beitrag gibt einen Überblick über die verschiedenen Arten von Reviews (Übersichtsarbeiten) als Formen der Evidenzsynthese mit besonderem Hinblick auf deren Stärken sowie Limitationen. Reviews können Wissen in aggregierter Form beschreiben und erlauben zusätzlich eine Bewertung der Studienqualität eingeschlossener Arbeiten. Die Aussagekraft bzw. die Vertrauenswürdigkeit der Ergebnisse eines Reviews hängt stark von der Qualität der eingeschlossenen Daten ab, weswegen eine konsequente Auswahlstrategie notwendig ist. Ein Basiswissen zur Literaturbewertung und zu möglichen Verzerrungseffekten ist auch in der Betrachtung von Übersichtsarbeiten notwendig. Zu diesem Zweck werden in diesem Artikel auch ausgewählte Werkzeuge zur Evidenzbewertung und zur Beurteilung des Biasrisikos vorgestellt.

## Abstract (en)

This manuscript outlines various types of review articles as forms of evidence synthesis with special regard to their strengths and limitations. Review articles not only present summarised data, but also offer an evaluation of the quality of the individual studies included in it. The validity and the reliability of outcomes of reviews is strongly dependent on the quality of the data included. For this reason, a comprehensive literature selection process is paramount. Fundamental knowledge of bias and literature assessment is also necessary when reading reviews. This article presents selected tools for evidence appraisal and evaluation of bias risk.

## Einleitung

Jährlich werden mehr als 1 Mio. medizinische Arbeiten in Fachzeitschriften publiziert [1]. Auch in der Urologie werden z.B. jährlich rund 250 neue Publikationen zum Prostatakarzinom in MEDLINE publiziert [2]. Alle zu lesen, ist unmöglich. Möchte man aber zu einer definierten Fragestellung einen aktuellen Überblick über die vorhandene relevante Datenlage erlangen, so können hochwertige systematische Übersichtsarbeiten den verlässlichsten Überblick verschaffen. Diese sog. Reviews können Wissen aus einzelnen Studien aggregiert darstellen. Mit der daraus resultierenden Ergebnisbasis können diese Fragestellungen meist mit einer größeren Aussagekraft beantwortet werden [3]. Sie beinhalten häufig auch eine Bewertung der Studienqualität eingeschlossener Arbeiten.

Hochwertige systematische Übersichtsarbeiten können den verlässlichsten Überblick verschaffen

Nach ihrem Schwerpunkt und ihrer Methodik unterscheiden wir verschiedene Arten von Reviews: narrative, systematische, Rapid, Living sowie Umbrella Reviews. Es ist wichtig, als Leser die Stärken und Limitationen solcher Übersichtsarbeiten zu kennen, um ihre Aussagekraft bestmöglich in Kontext setzen zu können.

## Hintergrund

Jeder medizinischen Handlung sollten die Prinzipien der evidenzbasierten Medizin (EbM) zugrunde liegen. Der rote Faden der EbM ist die Frage, die sich Ärzte beständig stellen: Woher wissen wir, ob eine bestimmte Therapie oder eine bestimmte Intervention wirksamer als eine andere ist? Die Zeiten, in denen ein Mediziner sein Wissen durch die Erfahrung seiner Jahre erwarb, gehören der Vergangenheit an. Heutzutage sind klinische Expertise, die Wünsche und Erwartungen der Patienten sowie die kumulierte wissenschaftliche Faktenlage die Basis der evidenzbasierten Entscheidungsfindung.

Die inhärente Abhängigkeit von Evidenz beinhaltet aber auch einige Fallstricke, da wissenschaftliche Aussagen von potenziellen Störfaktoren methodischer und individueller Natur beeinflusst werden können [4]. Nicht alle diese Störfaktoren können vollständig ausgeschaltet werden. Umso mehr ist es notwendig, als Leser jene Faktoren zu identifizieren und in Kontext setzen zu können, bevor eigenes Handeln dadurch geleitet wird. Die Menge an verfügbaren wissenschaftlichen Veröffentlichungen kann jedoch die Kapazitäten klinisch tätiger Mediziner überanstrengen (Informationsflut).

Evidenzsynthesen, also Zusammenfassungen von wissenschaftlichen Erkenntnissen in Form von systematischen Übersichtsarbeiten oder Reviews, bieten hier eine Möglichkeit, auf einem Blick die relevantesten Ergebnisse zu einer definierten Fragestellung zu erfahren [5]. Nicht nur bei Einzelstudien sondern auch bei Evidenzsynthesen kommt es auf die Qualität an: Die Qualität der zusammengefassten Arbeiten muss systematisch geprüft werden, ist die resultierende Evidenz doch nicht mehr als die Summe des Einzelnen. Die Erstellung von systematischen Übersichtsarbeiten folgt dabei einer präzise definierten Methodik, um eine verlässliche Aussagekraft der Ergebnisse zu gewährleisten.

Gut konzipierte und durchgeführte Studien zeichnen sich dadurch aus, dass systematische Fehler weitestmöglich identifiziert und möglichst auch ausgeglichen werden. Dadurch lässt sich eine Annäherung an den wahren Effekt darstellen, ohne falschen Schlüssen zu viel Raum zu geben. Gelingt dies, so erlauben die Ergebnisse Aussagen mit hoher Vertrauenswürdigkeit in die Evidenz („level of evidence“, LoE), wonach besonders in Leitlinienprogrammen graduiert wird.

Die verschiedenen Reviewformen werden im Folgenden näher erläutert:

## Narratives Review

Ein narratives Review besteht aus einer Akkumulation von Evidenz, die selektiv von den Autoren zu einem definierten Thema zusammengetragen wird. Diese Form der Evidenzsynthese hat den Vorteil, einen Überblick über ein Thema zu bieten, sie stellt allerdings eine Expertenmeinung dar [6].

Die Literaturauswahl für ein narratives Review obliegt dem Verfasser und orientiert sich nach der Ausrichtung des Artikels. Im Gegensatz zu systematischen Reviews, bei denen am Beginn der Arbeit eine klare Suchstrategie, möglicherweise unter Einbeziehung von Informationsspezialisten, definiert wird, ist eine solche strukturierte Literaturakquise nicht zwingend notwendig.

Allerdings kann in narrativen Reviews keine quantitative Zusammenfassung von Punktschätzern erfolgen (z. B. die Berechnung einer übergreifenden „gepoolten“ Odds Ratio aus den Odds Ratios von Einzelstudien; [7]). Weiterhin bleiben Bias und Studienqualität der Einzelstudien möglicherweise unzureichend berücksichtigt. Die subjektiven Einschätzungen der Autoren spielen eine wesentliche Rolle [8].

## Systematisches Review

Systematische Übersichtsarbeiten („systematic reviews“) sind zusammenfassende und qualitätsbewertende Darstellungen von Studienergebnissen (Tab. 1; [7]). Das Ziel ist hierbei, alle Studiendaten aus allen relevanten Primärstudien zu erfassen. Dabei können fakultativ Metaanalysen enthalten sein [9]. Die eingeschlossenen Studienergebnisse können dabei sowohl Interventions- als auch Beobachtungsstudien darstellen [10].

**Table 1:** 

Strukturierte Literaturrecherche	
Systematischer Einschluss geeigneter Arbeiten	
Narrative Zusammenfassung der verfügbaren Evidenz	
Bewertung der Evidenzqualität	
*Quantitatives Review*	Pooling von Effektschätzern
	Evaluation der Heterogenität gepoolter Schätzer
	Subgruppenanalysen
	Sensitivitätsanalysen
	Publikationsbias

Der Vorteil eines systematischen Reviews ist die systematische und strukturierte Erfassung sowie Bündelung der gesamten verfügbaren Evidenz zu einem Thema. Hierbei sollen alle relevanten Datenbanken, Studienregister und Publikationstypen eingeschlossen werden. Der methodisch strengste Ansatz sieht ein Vier-Augen-Prinzip bei der Selektion der Literatur, der Datenextraktion und der Studienbewertung vor, welche unabhängig voneinander stattfindet.

Die Methodik der Erstellung systematischer Reviews ist selbst Gegenstand wissenschaftlicher Arbeiten, Experten werden in Workshops und Kursen geschult [6]. In Deutschland bietet z. B. die Cochrane Collaboration solche Weiterbildungen an: https://www.cochrane.de/de/veranstaltungen.

Wie beim narrativen Review sind die Ergebnisse bzw. die Aussagekraft ebenfalls abhängig von der Menge und Qualität der zur Verfügung stehenden Einzelpublikationen. Allerdings bietet sich hier die Möglichkeit, die Qualität der eingeschlossenen Studien systematisch zu bewerten und möglicherweise Anpassungen für Qualitätsunterschiede und Bias bei der Ergebnisinterpretation vorzunehmen [7].

Metaanalysen in systematischen Reviews können über die Daten der Einzelstudien hinaus durch mathematische Modelle Evidenz generieren, wie etwa übergreifende Punktschätzer bei gepoolten Daten [7]. Allerdings sind derartige Möglichkeiten bedingt durch Qualität, Quantität und Homogenität der Datengrundlage der Studien. Nicht immer gelingt eine aussagekräftige Metaanalyse.

Nicht immer gelingt eine aussagekräftige Metaanalyse

Ist die Menge verfügbarer Einzelpublikationen jedoch groß, birgt auch dies potenzielle Nachteile: Die Sichtung einzelner Arbeiten, die Extraktion ihrer Ergebnisse und deren Zusammenfassung und Bewertung lässt die Erstellung eines qualitativ hochwertigen systematischen Reviews sehr umfangreich werden. Am Ende entsteht ein Gesamtbild über die Evidenz, wobei die Ergebnisse auf der Evidenzqualität und Sicherheit der Evidenz basieren. Sind diese limitiert, können daraus keine starken Empfehlungen abgeleitet werden.

## Sonderform Rapid Review

Hierbei handelt es sich um eine „schnelle“ Zusammenfassung verfügbarer Evidenz, bei deren Erstellung jedoch ebenfalls bestimmte methodische Regeln beachtet werden sollten [11]. Hilfreich sind solche Arbeiten besonders in Gebieten sich schnell entwickelnder Evidenz, wie beispielsweise der medikamentösen Tumortherapie des Nierenzellkarzinoms oder die SARS-CoV-2-Forschung („severe acute respiratory syndrome coronavirus type 2“). Allerdings bleibt festzustellen, dass der Begriff des Rapid Reviews noch recht jung ist und Definitionen wie Methodik sich erheblich unterscheiden können [12, 13]. Die Cochrane Collaboration selbst betreibt eine Arbeitsgruppe zur Methodendefinition von Rapid Reviews (https://methods.cochrane.org/rapidreviews/welcome).

Die potenziellen Nachteile dieser Form der Evidenzsynthese liegen auf der Hand: Schnelligkeit und Aktualität der Evidenzübersicht führen notwendigerweise zu Einbußen in der Methodik, der Qualität und der Tiefe der Zusammenfassung. Die zeitliche Limitation eines Rapid Reviews erlaubt meist auch nur eine weniger umfassende Literatursuche. Statt dem beim systematischen Review üblichen Vier-Augen- wird meist ein Zwei-Augen-Prinzip angewendet. Diese Kompromisse bergen auch ein Risiko, dass durch die limitierte Literatursuche gewisse Effekte unterschätzt werden.

## Sonderform Living Review

Living (systematic) Reviews sind Übersichtsarbeiten, welche kontinuierlich oder im Intervall ergänzt oder überarbeitet werden [14]. Neue Publikationen werden sofort in die bestehende Arbeit aufgenommen bzw. ergänzt, um die aktuellsten Informationen zu bieten. Sie unterscheiden sich in ihrer Methodik nicht wesentlich von systematischen Reviews. Analog zu sog. „living guidelines“ finden sie dort Anwendung, wo in kurzer Zeit große Mengen an neuer Evidenz entstehen und klassische Übersichtsarbeiten rasch obsolet werden. Die Cochrane Collaboration hat bisher sechs Living-systematic-Reviews veröffentlicht [15].

Living Reviews erfordern von ihren Erstellern eine besondere Anstrengung bezüglich Motivation und Arbeitsaufwand. In einer Evaluierung von beteiligten Autoren und Editoren wurde die andauernde Auseinandersetzung mit dem Reviewthema als besonders anspruchsvoll wahrgenommen [16].

## Sonderform Umbrella Review

Ein Umbrella Review wird auch „review of reviews“ genannt und ist eine systematische Zusammenfassung von Übersichtsarbeiten [17]. Üblicherweise wird damit eine Fragestellung oder ein medizinisches Problem beleuchtet, zu dem mehrere Interventionen beschrieben wurden, von welchen sich aber noch keine als überlegen herausgestellt hat. Bei Umbrella Reviews werden keine Einzelarbeiten überprüft, sondern nur Daten aus Reviews und Metaanalysen übernommen [18]. Wie auch bei den anderen Reviewformen sollte eine Qualitätsbewertung der eingeschlossenen Arbeiten erfolgen.

## Was macht ein qualitativ hochwertiges Review aus?

Vor einer eingehenderen Beschäftigung mit systematischen Reviews lohnt sich, in Erinnerung zu rufen, welche Fragestellungen mit diesen Evidenzsynthesen beantwortet werden können und welche nicht (Tab. 2).

**Table 2:** 

Was kann ein systematisches Review	Was kann ein systematisches Review nicht
Darstellung der aktuellen Evidenzlage	Generierung quantitativer Ergebnisse bei einer nur geringen Zahl eingeschlossener Studien
Bewertung der Studienqualität der eingeschlossenen Arbeiten	Zusammenfassung heterogener Studiendaten
Erstellung gepoolter Schätzer (wenn möglich)	Adjustierung für Störgrößen, die in den einzelnen eingeschlossenen Studien nicht berücksichtigt wurden
Untersuchung von Subgruppen und Interaktionen	Evaluation von Primärdaten der einzelnen Studien (außer in gepoolten Metaanalysen)
Analyse der Evidenzentwicklung über die Zeit (kumulative Metaanalyse)	
Nachweis von Publikationsbias	
Vergleich mehrerer Interventionen (Netzwerkmetaanalyse)	
Dosis-Wirkungs-Beziehungen (in Subgruppenanalysen)	
Zusammenfassung von Evidenz für seltene Erkrankungen oder kleine Effekte oder wenn die Effektschätzer von Primärstudien unterschiedliche Ergebnisse aufweisen	

Bestimmte grundlegende Rahmenbedingungen in der Erstellung und Bewertung systematischer Reviews haben sich etabliert. Die Cochrane Collaboration hat für die Beantwortung wissenschaftlicher Fragen ein mehrstufiges Programm erarbeitet [7]:

1. klinische Fragestellung,
2. Literatursuche,
3. Bewertung der Qualität der Literatur,
4. Zusammenfassung der Evidenz,
5. Interpretation der Ergebnisse.

Je genauer und spezifischer die klinische Fragestellung und Erfassung der möglichen Probleme, je eindeutiger die Reviewfrage, desto definierter kann die Literatursuche erfolgen.

Die Reviewfrage wird hierbei ebenfalls durch ein Rahmenwerk PICO („patient, intervention, control/comparison und outcome“) vorgegeben [19]:

- P – Um welche Population handelt es sich?
- I – Welche Intervention wird durchgeführt?
- C – Wie sieht der „comparison“/Vergleich zur Kontrollgruppe aus?
- Welche relevanten Endpunkte („outcome“) werden erreicht?

Zusätzlich stellt sich die Frage des zu untersuchenden Zeitraums, der Mindestbeobachtungszeit, des Studiendesigns und -settings.

Die Aussagekraft einer Übersichtsarbeit hängt von der Qualität der zugrunde liegenden Evidenz ab

Die Basis eines systematischen Reviews bildet die eingeschlossene Literatur anhand der klinischen Fragestellung. Nur wenn der Erkenntnisstand zum Zeitpunkt der Literatursuche akkurat abgebildet wird, kann ein valider Überblick entstehen. Deren Identifikation erfolgt nach strengen Regeln: Eine Literaturrecherche sollte die wichtigsten Literaturdatenbanken wie z. B. EMBASE, MEDLINE und die Cochrane Library umfassen [6]. Für jede einzelne Datenbank wird ein Suchalgorithmus festgelegt, in der später inklusive Suchdatum und Zahl der gefundenen Publikationen dokumentiert wird. Dies garantiert die Reproduzierbarkeit. Leider werden nicht alle Forschungsergebnisse publiziert oder in den bekannten Literaturdatenbanken indexiert, weswegen sich eine umfassende Beschäftigung mit der Datenlage auch auf Studienregister und sog. „graue Literatur“ ausweiten muss.

Prinzipiell hängt die Aussagekraft der Übersichtsarbeit entscheidend von der Qualität der zugrunde liegenden Evidenz ab. Daher sollte diese für jede eingeschlossene Publikationen einzeln bewertet werden. Hierzu stehen verschiedene Instrumente (je nach Studiendesign) zur Verfügung: z. B. das neue *Risk-of-bias- (RoB-2-)Tool *der Cochrane Collaboration zur Bewertung von randomisierten klinischen Studien, die *Downs-and-black-Checkliste* zur Bewertung von randomisierten und nicht-randomisierten klinischen Studien, das *ROBINS-I-Tool *(„risk of bias in non-randomised studies of interventions“) zur Bewertung von nicht-randomisierten Studien oder die* Newcastle Ottawa *Scale für Beobachtungsstudien [20–23].

Kritiker werfen selbst den etabliertesten dieser Werkzeuge vor, uneinheitliche Ergebnisse zu generieren [24]. So bleibt die Evaluierung der Studienqualität eine subjektive Einschätzung des Autorenteams, die nicht vollständig objektivierbar ist. Gemeinsam ist allen Instrumenten jedoch die Untersuchung mehrerer Bereiche der Studienqualität (z. B. des Biasrisikos).

## Bewertungstools: „risk of bias“ und PRISMA

### Evaluation des Biasrisikos

Die Aussagekraft wissenschaftlicher Studien hängt wesentlich davon ab, wie diese geplant, durchgeführt und deren Ergebnisse interpretiert werden. In einem multifaktoriellen Umfeld wie in der Medizin ist es meist schwierig, den „reinen“ Effekt einer Intervention oder einer Beobachtung darzustellen. Werden bekannte und unbekannte Störfaktoren nicht adäquat begegnet, kann es leicht zu einer falschen Einschätzung des Effekts kommen [25]. Es ist daher wichtig, systematische Störfaktoren („confounder“), das Potenzial auf Zufallsfehler oder Gründe für eine Verzerrung („bias“) der Ergebnisse zu kennen und in die Ergebnisinterpretation einfließen zu lassen. Diese Limitationen in Studien zu verstehen, ermöglicht uns, die Aussagekraft der Arbeit korrekt einzuschätzen.

Confounder, sofern bekannt, müssen im Studiendesign berücksichtigt werden, z. B. durch Einteilung in Gruppen. Hier bietet sich das bekannte Beispiel der Korrelation von Kaffeekonsum und kardiovaskulärem Risiko an: Ohne das Wissen, dass Raucher auch häufiger Kaffee trinken und Rauchen ein unabhängiger Risikofaktor für kardiovaskuläre Erkrankungen ist, würden hier falsche Schlüsse gezogen. Unbekannte Confounder müssen über eine adäquate Randomisierung kontrolliert werden.

Zufallsfehler treten dann auf, wenn durch Zufallseffekte ein eigentlicher Populationseffekt in einer Studie nicht darstellbar wird. Der Effekt des Zufalls kann durch eine ausreichend große Studienpopulation kontrolliert werden, auch wenn keine pauschale Mindestgröße definiert werden.

Bias sind systematische Fehler in der Studienkonzeption, -durchführung und -auswertung

Bias sind systematische Fehler in der Studienkonzeption, -durchführung und -auswertung. Das Vorliegen von Bias kann in der Betrachtung einer Studie nicht direkt gemessen werden, sondern nur über das Studiendesign und -protokoll evaluiert werden. Häufige Arten des Bias (und Maßnahmen zur Vermeidung) werden in Tab. 3 angeführt. Ein neues und interaktives Tool zur Bewertung der Berichtserstellung und -erfassung ist das ROB-ME-Tool („risk of bias due to missing evidence“), welches aktuell unter https://www.riskofbias.info/welcome als Pilotprogramm abzurufen ist (s. Infobox 1).

**Table 3:** 

Bias	Definition	Maßnahmen zur Verhinderung
*Selection bias*	Systematische Unterschiede zwischen Teilnehmergruppen in der Empfänglichkeit für eine Intervention	Strikte Rekrutierungsregeln
		Randomisierung der Gruppen
		Ausreichende Größe der Studienpopulation
		Studienteilnehmer aus derselben Population rekrutieren
*Performance bias*	Systematische Unterschiede zwischen Teilnehmergruppen in der Betreuung der StudienteilnehmerInnen	Standardisiertes Studienprotokoll verwenden
		Verblindung der Probanden und Untersucher
		Unabhängige Untersucher
*Detection bias*	Systematische Unterschiede zwischen Teilnehmergruppen in der Erhebung des Endpunkts/Expositionen	Standardisiertes Erhebungsprotokoll verwenden
		Objektive, homogene Definition von Outcomes
		Verblindung der Probanden und Untersucher
*Attrition bias*	Systematische Unterschiede zwischen Teilnehmergruppen, welche zu verfrühtem Ausscheiden aus einer Studie führen	Genaue Darstellung der Aussteiger und Ausstiegsgründe
		Intention-to-treat- (ITT-)Analyse
*Language/geographical bias*	a. Bevorzugte Indexierung von Publikationen bestimmten Sprachräumen b. Fehlende Möglichkeit zur Indexierung in Entwicklungsländern	Durchsuchen von anderssprachigen Literaturquellen
*Publication/reporting bias*	a. Unpublizierte Daten sind nicht/schwer zu finden b. Negative Endpunkte werden weniger oft publiziert	Suche in Studienregistern
		Suche nach unpublizierten Studienberichten
*Retrieval bias*	Unvollständige Identifizierung der Studien kann die aktuelle Erkenntnislage beeinflussen	Literaturrecherche systematisch halten, um alle Studien zu erfassen
*Citation bias*	Positive und signifikante Studienergebnisse werden häufiger zitiert	Literaturrecherche systematisch halten, um alle Studien zu erfassen

#### Infobox 1 QR-Codes


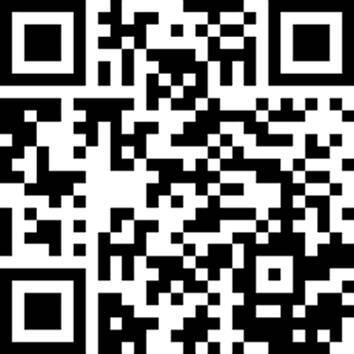


QR-Code 1: https://www.riskofbias.info/welcome/rob-me-tool als interaktives Tool zur Erfassung und Bewertung von Bias
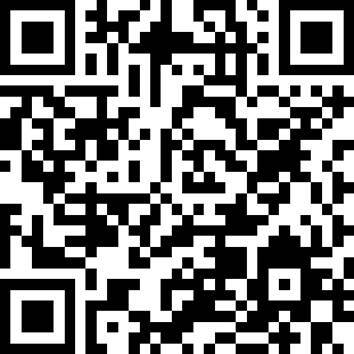


QR-Code 2: Eine interaktive Version zu Abb. 3 kann personalisiert abgerufen werden unter https://github.com/nealhaddaway/SRflowdiagram/blob/main/README.md (Abruf 28.12.2020)
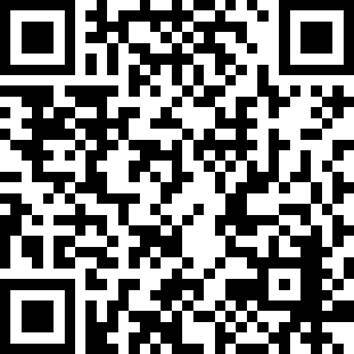


QR-Code 3: PRISMA 2020: updated guidelines for reporting systematic reviews and meta-analyses https://www.youtube.com/watch?v=Y-fu00PSm9o (Abruf 02.01.2021)

**Figure 1:**
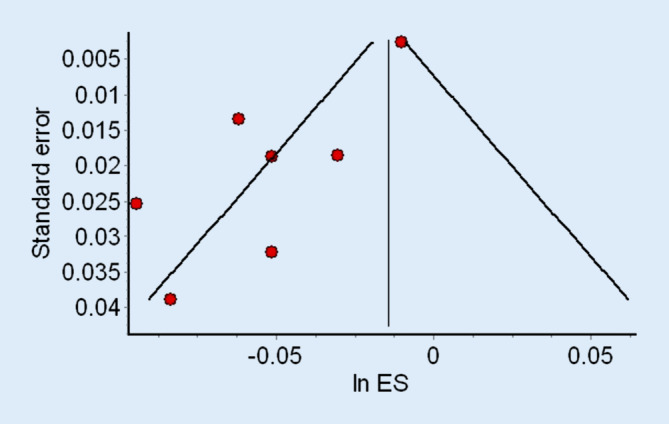

Der Publikationsbias kann in einem systematischen Review quantifiziert werden: Hierzu wird üblicherweise aus den Punktschätzern der einzelnen, in das systematische Review eingeschlossenen Studien ein sog. Funnel Plot generiert (Abb. 1; [7]).

Dieser Graph kann optisch auf eine Asymmetrie untersucht werden, welche wiederum auf einen Publikationsbias hinweist. Auch statistische Tests wie der Begg’s [28] oder Egger’s Test [29] können einen Publikationsbias nachweisen. Für alle Verfahren ist allerdings eine größere Anzahl an in das systematische Review eingeschlossenen Einzelstudien notwendig: Ein Funnel Plot verlangt ca. 10 Studien, der Egger’s Test weist meist erst ab 20 Studien eine adäquate Power auf. In kleinen systematischen Reviews kann daher keine Beurteilung eines Publikationsbias stattfinden [7, 30, 31].

Neben einer deskriptiven Ergebnisbeschreibung sollte, wenn möglich, eine quantitative Analyse der vorliegenden Evidenz erfolgen. Jedoch unterscheiden sich Generierung und Präsentation selbiger Evidenz in den einzelnen eingeschlossenen Studien oft erheblich. Daher gilt es, die Studienergebnisse aller eingeschlossener Arbeiten auf einen Nenner zu bringen. Dies geschieht durch eine strukturierte Erfassung der Inhalte nach einem a priori festgelegten Erhebungsplan. Für einen qualitativ hochwertige Analyse sollte dieser Vorgang durch mindestens zwei unabhängige und gegeneinander verblindete Autor erfolgen [7].

**Figure 2:**
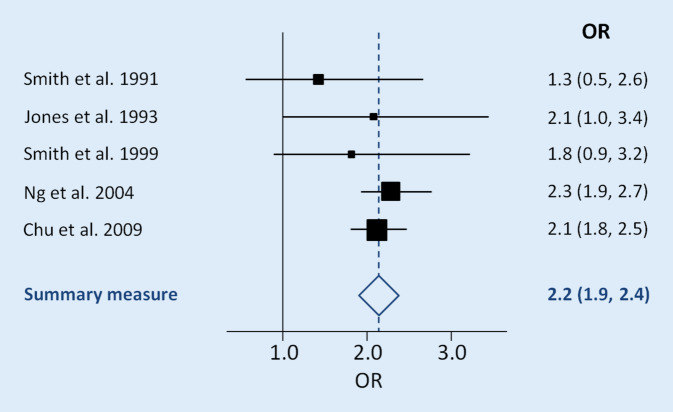

In einem systematischen Review inklusive Metaanalyse erfolgt ein sog. *Pooling* von Zahlenwerten: z. B. Odds Ratios oder Hazard Ratios inklusive ihrer Konfidenzintervalle und *p*-Werte werden zusammengefasst und ein gemeinsamer Schätzer berechnet. Abb. 2 zeigt exemplarisch einen sog. Forest Plot, der die Punktschätzer einzelner Studien zusammenfasst [7].

Ob eine Mindestanzahl von Studien erforderlich ist, um ein Pooling durchführen zu können, ist umstritten und wird kontrovers diskutiert: Bei wenigen Einzelstudien kann es zu verzerrten Effekten kommen [33].

Von einem gepoolten Schätzer muss die sog. Heterogenität bestimmt werden, welche in gewissem Maß eine Aussage über die Zuverlässigkeit des errechneten Schätzers ermöglicht [7]. Bei relevanter Heterogenität kann das Ergebnis der Analyse nicht für Schlussfolgerungen herangezogen werden, um die Ursache der Heterogenität zu ergründen. Sollte es die Größe der Metaanalyse erlauben, sind Subgruppenanalysen erforderlich. Diese sollten ebenfalls *a priori* festgelegt worden sein, damit die Analysen nicht durch die Ergebnisse beeinflusst werden („data dredging“; [7]). Bei hoher Heterogenität sollte hier keine quantitative Analyse erfolgen. Entsprechende „gescheiterte“ Analyseversuche sollten dennoch transparent beschrieben werden.

Von einem gepoolten Schätzer muss die sog. Heterogenität bestimmt werden

Je nach Poolingmethode haben Studien einen unterschiedlich großen Einfluss auf den gepoolten Schätzer. Um auszuschließen, dass nur Studien mit einem großen Einfluss auf den gepoolten Schätzer das Ergebnis bestimmen, werden Metainfluenzanalysen durchgeführt [34]. Hierbei werden Studien mit einem großen Einfluss ausgeschlossen und die Änderung des gepoolten Schätzers evaluiert.

Weiterhin gilt es zu untersuchen, ob sich die Evidenzlage über die Jahre geändert hat: In sog. kumulativen Metaanalysen werden einzelne Studien chronologisch nach Erscheinungsjahr in das Pooling eingeschlossen: So wird eine quantitative oder gar qualitative Änderung des gepoolten Schätzers über die Zeit ersichtlich [34].

Der Großteil quantitativer Reviews handelt Fragestellungen mit binären Interventionsparametern (z. B. Aspirin vs. kein Aspirin gegen Kopfschmerzen) ab. Allerdings sind auch Dosis-Effekt-Abschätzungen (z. B. die optimale Aspirin-Dosis) möglich [35]. Netzwerkmetaanalysen erlauben den Vergleich mehrerer Intervention (z. B. Aspirin vs. Ibuprofen vs. Novalgin vs. ein Phytotherapeutikum bei Kopfschmerzen; [7]). Für letztere Analysen ist jedoch eine ungleich größere Literaturgrundlage nötig. Auch hier steht und fällt die Vertrauenswürdigkeit bzw. Aussagekraft der Ergebnisse eines systematischen Reviews wieder mit der Zahl der eingeschlossenen Studien und deren Qualität.

### Berichtung eines systematischen Reviews: PRISMA

**Figure 3:**
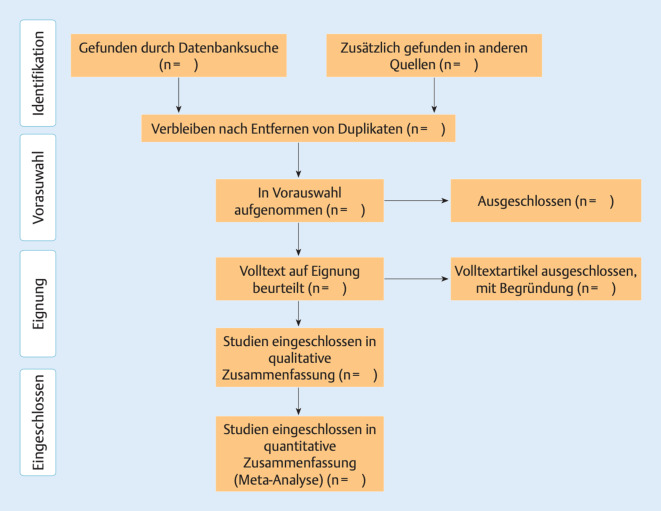

Ein weiteres Bewertungstool für die Qualität von Systematic Reviews und Metaanalysen ist PRISMA („preferred reporting items for systematic reviews and meta-analyses“, bevorzugte Berichtselemente für systematische Übersichten und Metaanalysen; [36]). Dieses beinhaltet ein Flussdiagramm zur Beschreibung der verschiedenen Phasen einer systematischen Übersicht (s. QR-Code 2 und Abb. 3). Dazu kommt eine Checkliste mit detaillierten Kriterien der einzelnen Publikationsabschnitte der zu bewertenden Arbeit von Einleitung bis Diskussion inklusive finanzieller Unterstützung. Die PRISMA-Checkliste wird beständig unter öffentlicher Diskussion überarbeitet (s. QR-Code 3 und Abb. 3; [37]). Diese neueste Version ist unter dem Titel „PRISMA 2020 explanation and elaboration“ als Preprint verfügbar [38]. Exemplarisch nennen wir hier die 12 wichtigsten Punkte für die Bewertung eines Abstracts (Tab. 4).

**Table 4:** 

1	Identifizieren Sie den Report als systematisches Review
2	Formulieren Sie die Zielsetzung oder Fragestellung des Reviews
3	Bestimmen Sie die Ein- und Ausschlusskriterien des Reviews
4	Geben Sie die Informationsquellen (z. B. Datenbanken, Register) an, die zur Identifizierung von Studien verwendet werden, sowie das Datum des letzten Datenabrufs
5	Spezifizieren Sie die Methoden zur Bewertung des Verzerrungspotenzials („risk of bias“) in den eingeschlossenen Studien
6	Geben Sie die Methoden an, mit denen die Ergebnisse präsentiert und zusammengestellt werden (z. B. Datentabellen und Forest Plots)
7	Geben Sie die Gesamtzahl der eingeschlossenen Studien und Teilnehmer an und fassen Sie die relevanten Merkmale der Studien zusammen
8	Beschreiben Sie die Hauptergebnisse, bevorzugt unter Nennung der Anzahl der eingeschlossenen Studien und Teilnehmer für jeden einzelnen Endpunkt. Wenn eine Metaanalyse durchgeführt wurde, geben Sie das Konfidenzintervall an. Beim Vergleich von Gruppen geben Sie die Richtung des Effekts an (z. B. welche Gruppe bevorzugt wird)
9	Geben Sie eine kurze Zusammenfassung der Einschränkungen der Evidenz (z. B. das „risk of bias“, Widersprüche und Ungenauigkeiten in der Studie)
10	Geben Sie eine allgemeine Interpretation der Ergebnisse und wichtigen Folgerungen
11	Geben Sie die Hauptfinanzierungsquelle für das Review an
12	Geben Sie den Registernamen und die Registrierungsnummer an

## Zusammenfassung

Systematische, qualitativ hochwertige Übersichtsarbeiten sind selbstständige Forschungsarbeiten, die mittlerweile als Originalarbeiten angesehen werden. Sie identifizieren systematisch relevante Studien, bewerten deren Ergebnisse und Qualität und fassen diese wissenschaftlich zusammen (Tab. 1). Systematische Übersichtsarbeiten bieten dadurch einem Fachpublikum die relevanten Ergebnisse zu einer vordefinierten Fragestellung, ohne sich mit einer größeren Anzahl einzelner Studien im Detail beschäftigen zu müssen. Immer mehr unserer Entscheidungsgrundlagen fußen auf den Erkenntnissen solcher systematischen Forschungsarbeiten. Je nach Umfang und Hintergrund der zu beantwortenden Fragestellung existieren verschiedene Formen der Evidenzsynthese, welche jeweils ihre systematischen Vorteile und Limitationen aufweisen: Analog der durch Dritte aufgearbeitete Information ist auch in der Betrachtung systematischer Übersichtsarbeiten die Kenntnis um Stärken, Limitationen und möglichen Störfaktoren wichtig. Insgesamt profitieren systematische Übersichtsarbeiten jedoch von zahlreichen systematisch-inhärenten Vorteilen gegenüber einzelnen Studien.

Je vollständiger die Evidenzbasis, desto sicherer ist das Ergebnis einer Evidenzsynthese

Kurz gesagt: Je vollständiger die Evidenzbasis, desto sicherer ist das Ergebnis einer Evidenzsynthese. Zu manchen Fragestellungen, bei denen wenig Evidenz oder Evidenz mangelnder Qualität vorhanden ist, wird das Ergebnis systematischer Übersichtsarbeiten zwar eine umfassendere, aber nicht zwingenderweise vertrauenswürdige Antwort hervorbringen.

## Fazit für die Praxis

- Systematische Übersichtsarbeiten identifizieren relevante Studien, bewerten deren Ergebnisse und Qualität und fassen diese systematisch zusammen.
- Je vollständiger die Evidenzbasis, desto vertrauenswürdiger das Ergebnis einer Evidenzsynthese.
- Eine systematische Literatursuche ist die Grundlage für die Vollständigkeit der Ergebnisse.
- Die Evidenz kann nicht nur rein beschreibend sondern auch quantitativ zusammengefasst werden
